# Benzo(a)pyrene Exposure Suppressed Osteogenic and Adipogenic Differentiation of Human Bone Marrow-Derived Mesenchymal Stem Cells and Was Associated with Senescence-like Response

**DOI:** 10.3390/ijms272110026

**Published:** 2026-11-09

**Authors:** Kanyaluck Jantakee, Duangrat Tantikanlayaporn, Chawalit Ngernsombat, Tanapan Siangcham, Kant Sangpairoj

**Affiliations:** 1 Research Unit in Nutraceuticals and Food Safety, Faculty of Medicine, Thammasat University, Pathumthani 12120, Thailand; kanyaluckjan@gmail.com; 2 Division of Cell Biology, Department of Preclinical Science, Faculty of Medicine, Thammasat University, Pathumthani 12120, Thailand; dkanlayaporn@gmail.com; 3 Division of Biochemistry, Department of Preclinical Science, Faculty of Medicine, Thammasat University, Pathumthani 12120, Thailand; ngechawa@tu.ac.th; 4 Faculty of Allied Health Sciences, Burapha University, Chonburi 20131, Thailand; tanapan@go.buu.ac.th; 5 Division of Anatomy, Department of Preclinical Science, Faculty of Medicine, Thammasat University, Pathumthani 12120, Thailand

**Keywords:** mesenchymal stem cell, benzo(a)pyrene, differentiation, senescence, bone, transcriptomic

## Abstract

Human exposure to benzo(a)pyrene [B(a)P] from contaminated environmental pollutants may induce cellular senescence. Mesenchymal stem cells (MSCs) are responsible for maintaining bone homeostasis, mineralization and integrity. Senescent MSCs may result from exogenous chemical exposure, leading to diminished osteogenic potential and increased bone loss. This study elucidated the toxicological effects of B(a)P exposure in human bone marrow-derived MSCs (BM-hMSCs) under induced osteogenic and adipogenic differentiation by in vitro testing and transcriptomic analyses. Cell viability assays following 1–20 µM of B(a)P showed no toxic effects after 24 to 72 h of exposure. Non-cytotoxic concentrations of B(a)P (1–20 µM) suppressed the differentiation of BM-hMSCs into osteogenic and adipogenic lineages over 7 to 21 days, as indicated by reduced phenotypic parameters and molecular expression of differentiation markers compared with differentiation-induced cells. B(a)P decreased osteoblastic proliferation of signaling *Wnt/β-catenin*, with increased *AhR/AhRR* expression, which is related to osteogenic damage. Furthermore, B(a)P increased the expression of senescence markers, includingintracellular β-galactosidase, *p21* and the secretory markers IL-1β and IL-6, in osteogenic-differentiated BM-hMSCs. The transcriptomic profile of osteogenic-differentiated BM-hMSCs exposed to 10 µM of B(a)P for 21 days revealed upregulation of stress and inflammatory responses, along with downregulation of cell cycle regulation and genes associated with cellular integrity. RNA-seq profiles also revealed upregulation of senescence-associated markers. Collectively, B(a)P may impair their differentiation capacity and is associated with senescence induction, partly through AhR activation. This study highlights the implications for skeletal health risk of chronic exposure to environmental pollutants by detection of senescent-associated markers.

## 1. Introduction

Benzo(a)pyrene [B(a)P], a prototypical polycyclic aromatic hydrocarbon (PAH), is ubiquitously generated from incomplete combustion of organic matter and is classified by the International Agency for Research on Cancer (IARC) as a Group 1 human carcinogen [1]. Human exposure occurs primarily via inhalation of polluted air, ingestion of contaminated food, and dermal contact, with fine particulate matter (PM2.5) serving as a major airborne carrier [1,2]. Beyond its well-established carcinogenicity, accumulating evidence demonstrates that B(a)P exerts significant osteotoxic effects, including reduced bone mass, impaired osteoblast differentiation, and altered bone remodeling, partly through hormonal disruption and epigenetic reprogramming [3,4].

Human bone marrow-derived mesenchymal stem cells (BM-hMSCs) are multipotent progenitors critical for bone homeostasis, fracture repair, and the hematopoietic niche [4,5]. Their osteogenic differentiation is governed by the *BMP/SMAD*, *TGF-β*, and *Wnt/β-catenin* signaling pathways, with the master transcription factors *RUNX2* and *osterix* acting as key downstream effectors [6]. B(a)P interferes with this process primarily by activating the aryl hydrocarbon receptor (*AhR*), which downregulates *BMP-2* signaling via reduced *BMPR-II* expression and suppresses *TGF-β1/SMAD4* and *ERK/AKT* pathways, resulting in impaired self-renewal and attenuated osteogenic potential [7,8]. Studies using AhR ligands further confirm that *AhR* transactivation consistently suppresses alkaline phosphatase (ALP) activity, matrix mineralization, and key osteogenic markers, including DLX5 and OPN in primary BM-hMSCs [9,10]. B(a)P has been shown to inhibit human MSC-derived adipogenesis via activation of the aryl hydrocarbon receptor (AhR), resulting in reduced lipid accumulation and downregulation of adipogenic markers such as fatty acid binding protein-4 (FABP4) [11]. Mechanistically, AhR signaling has been reported to interfere with PPARγ-driven adipogenic programs, further supporting its role as a negative regulator of adipocyte differentiation [12]. Cellular senescence of MSCs is increasingly recognized as a driver of age-related bone loss [13]. Senescent MSCs exhibit cell cycle arrest, elevated senescence-associated secretory phenotype (SASP) factors, and markedly diminished osteogenic potential [14,15]. DNA damage triggers the ATM/p53/p21 axis, promotes mitochondrial dysfunction, and amplifies reactive oxygen species (ROS), establishing a self-reinforcing senescent state [15]. B(a)P is known to induce replicative senescence, DNA strand breaks, and elevated p21 expression in stem cells and other cell types, suggesting that it may similarly drive an MSC aging phenotype [14,16]. The resulting SASP, comprising IL-6, IL-8, TNF-α, and matrix metalloproteinases, disrupts the bone marrow niche and promotes a pro-osteoclastic, anti-osteoblastic environment [17].

Despite these advances, how B(a)P specifically affects proliferation, osteogenic differentiation, and cellular senescence in BM-hMSCs as an integrated process remains poorly defined. Recent work highlights metabolic dysregulation, epigenetic reprogramming, and mechanotransduction as key determinants of MSC senescence [17], yet these mechanisms have not been examined in the context of B(a)P-induced osteotoxicity. Therefore, this study aims to investigate the effects of B(a)P on BM-hMSC proliferation, osteogenic differentiation, and senescence, with emphasis on AhR- and DNA-damage-linked mechanisms, to provide mechanistic insights that may inform targeted rejuvenation strategies in pollutant-exposed skeletal tissue.

## 2. Results

### 2.1. Cytotoxicity Effect of B(a)P

**Figure 1:**
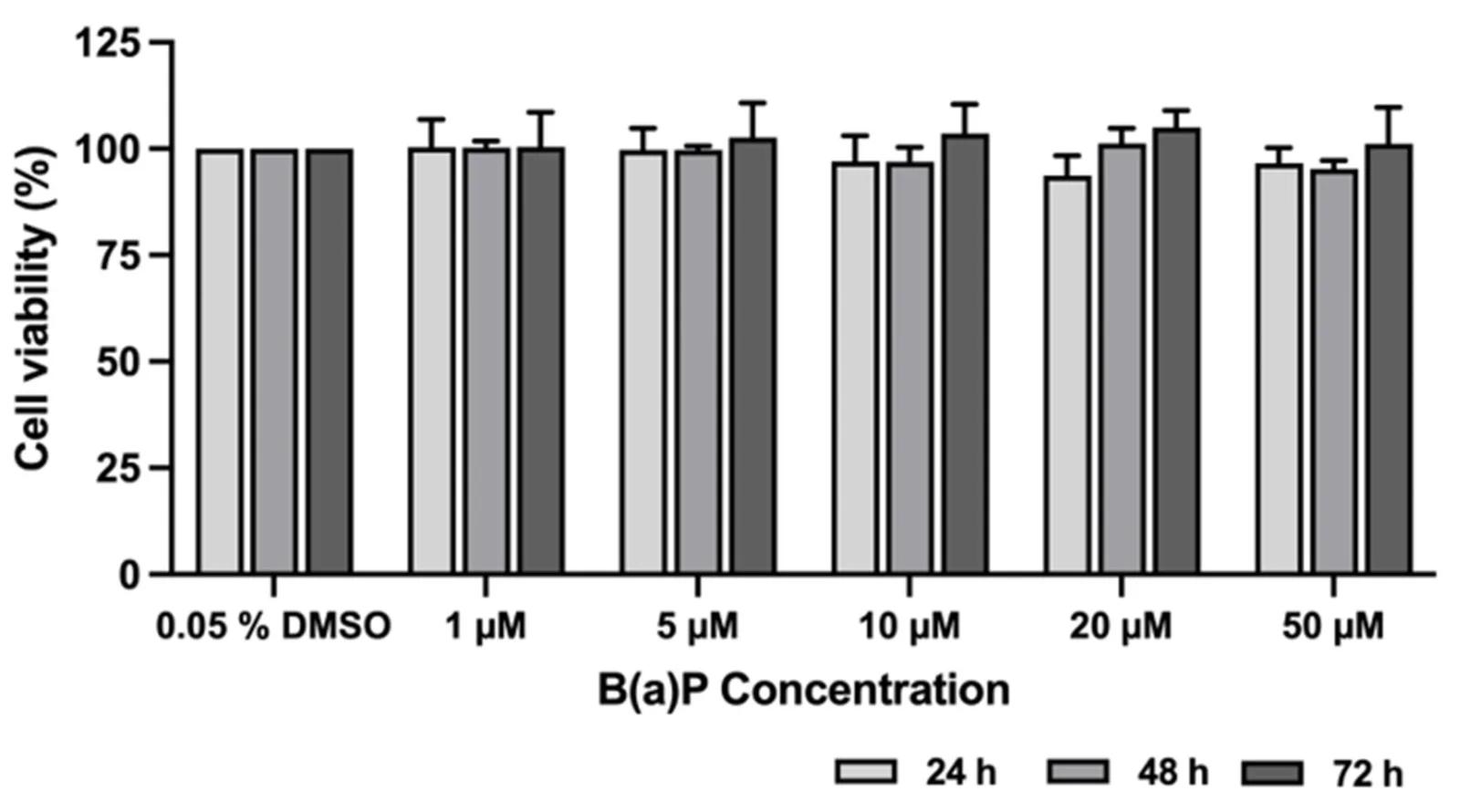
The cytotoxicity effect of B(a)P on BM-hMSCs cells. The compounds were treated at different concentrations of 1–50 μM as B(a)P. The percentage of cell viability was elucidated using MTT assay, which showed cell viability relative to the non-treatment control. The results show mean ± SD values (*n* = 3).

The cytotoxicity of B(a)P inBM-hMSCs was first assessed by the MTT viability assay after exposure for 24, 48, and 72 h. The viability of B(a)P-exposed cells remained unchanged over 24–48 h and increased insignificantly at 72 h post-exposure. Cell viability remained above 80% at each time point compared to the control (Figure 1). These result indicated that B(a)P did not cause cytotoxicity in BM-hMSCs by retaining cell viability within 72 h.

### 2.2. Detection of Senescence-Associated β-Galactosidase in BM-hMSCs Following B(a)P Exposure

**Figure 2:**
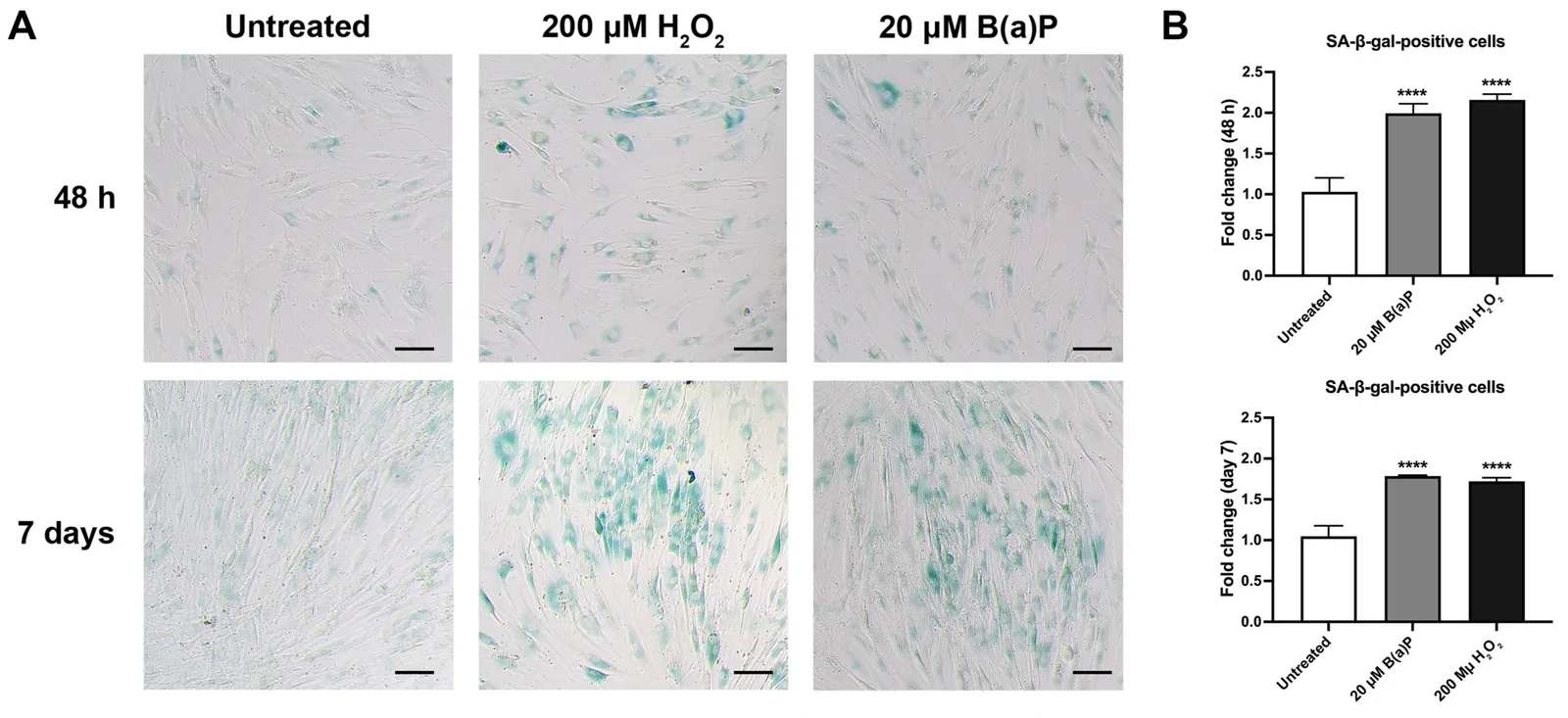
Expression of senescence-associated β-galactosidase following B(a)P exposure on BM-hMSCs. (**A**) Representative photomicrographs of BM-hMSCs stained with SA-β-galactosidase detection assay after exposure to 20 µM of B(a)P at 48 h and 7 days compared with H_2_O_2_-exposed cells as a positive control. Blue-stained cells indicate β-galactosidase accumulation. (**B**) The relative fold change in SA-β-gal-positive cells was calculated relative to the untreated control. The results show mean ± SD values (*n* = 3) (**** *p*  <  0.0001).

The presence of senescence-associated β-galactosidase (SA-β-gal) in BM-hMSCs following B(a)P exposures was assessed to identify senescence-associated phenotypes at 48 h and 7 days post-exposure. At 48 h of post-exposure to 20 µM of B(a)P, mild SA-β-gal staining was visualized as shown in dark blue-stained cells resembling those in the H_2_O_2_-exposed group, which served as a positive control. In contrast, the untreated control showed pale blue staining. At 7 days post-exposure, the group exposed to 20 µM of B(a)P-showed intensely blue-stained cells, indicating increased β-galactosidase expression (Figure 2A). Quantitative analysis of SA-β-gal activation further revealed a significant increase in the relative fold change of SA-β-gal-positive cells after 48 h and 72 h of B(a)P exposure, reaching 1.99 ± 0.12-fold and 1.79 ± 0.01-fold, respectively, compared with the untreated control (Figure 2B). These results suggest that B(a)P may promote a senescence-associated phenotype in BM-hMSCs.

### 2.3. BM-hMSCs Differentiation into Adipocytes Under Adipogenic Differentiation Conditions

**Figure 3:**
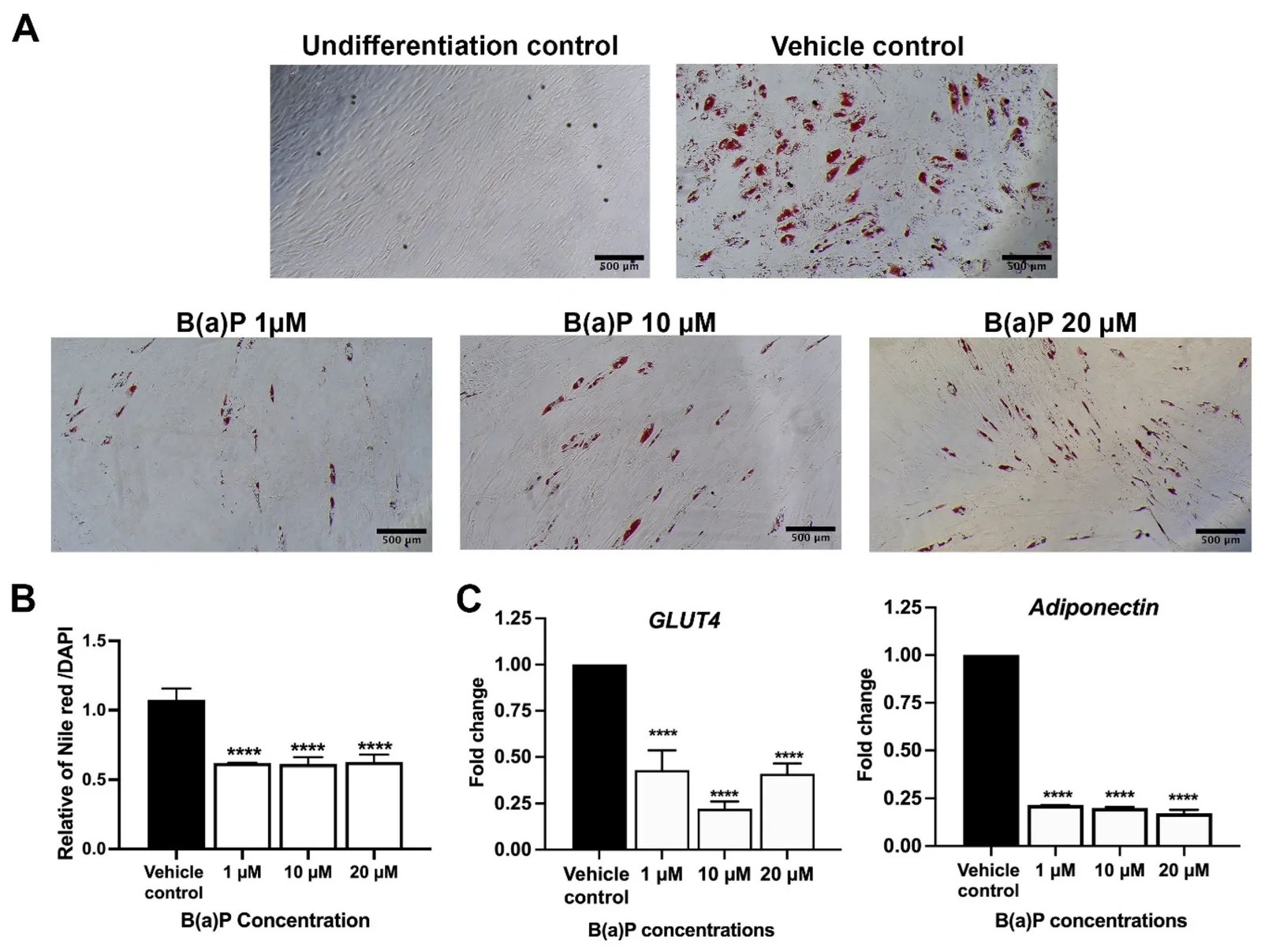
The effect of B(a)P on adipogenic differentiation of BM-hMSCs. (**A**) Adipocyte-like differentiating cells were stained with Oil Red O. (**B**) Adipocyte differentiation was quantified using Nile red staining, and it indicated that B(a)P suppressed adipogenic differentiation. (**C**) The relative gene expression of GLUT4 and adiponectin was measured by qPCR. The data are presented as mean  ±  SD (*n* = 3). **** *p*  <  0.0001). The scale bar represents 500 μm at 10× magnification.

To evaluate the effect of B(a)P on adipogenic differentiation of BM-hMSCs, cells were treated with 1, 10, and 20 μM B(a)P under the induction of stem cell differentiation into adipocytes. The results revealed that B(a)P at low concentrations reduced adipogenic differentiation compared with the adipogenic differentiation control group at 14 days, as shown by Nile red staining (Figure 3A,B). Furthermore, the expression of adipogenic marker genes GLUT4 and adiponectin was decreased when compared with the differentiation control (Figure 3C). These results suggest that B(a)P may affect the expression of adipogenic genes and reduce the efficacy of adipocyte differentiation.

### 2.4. BM-hMSCs Differentiation into Osteoblasts Under Osteogenic Conditions

**Figure 4:**
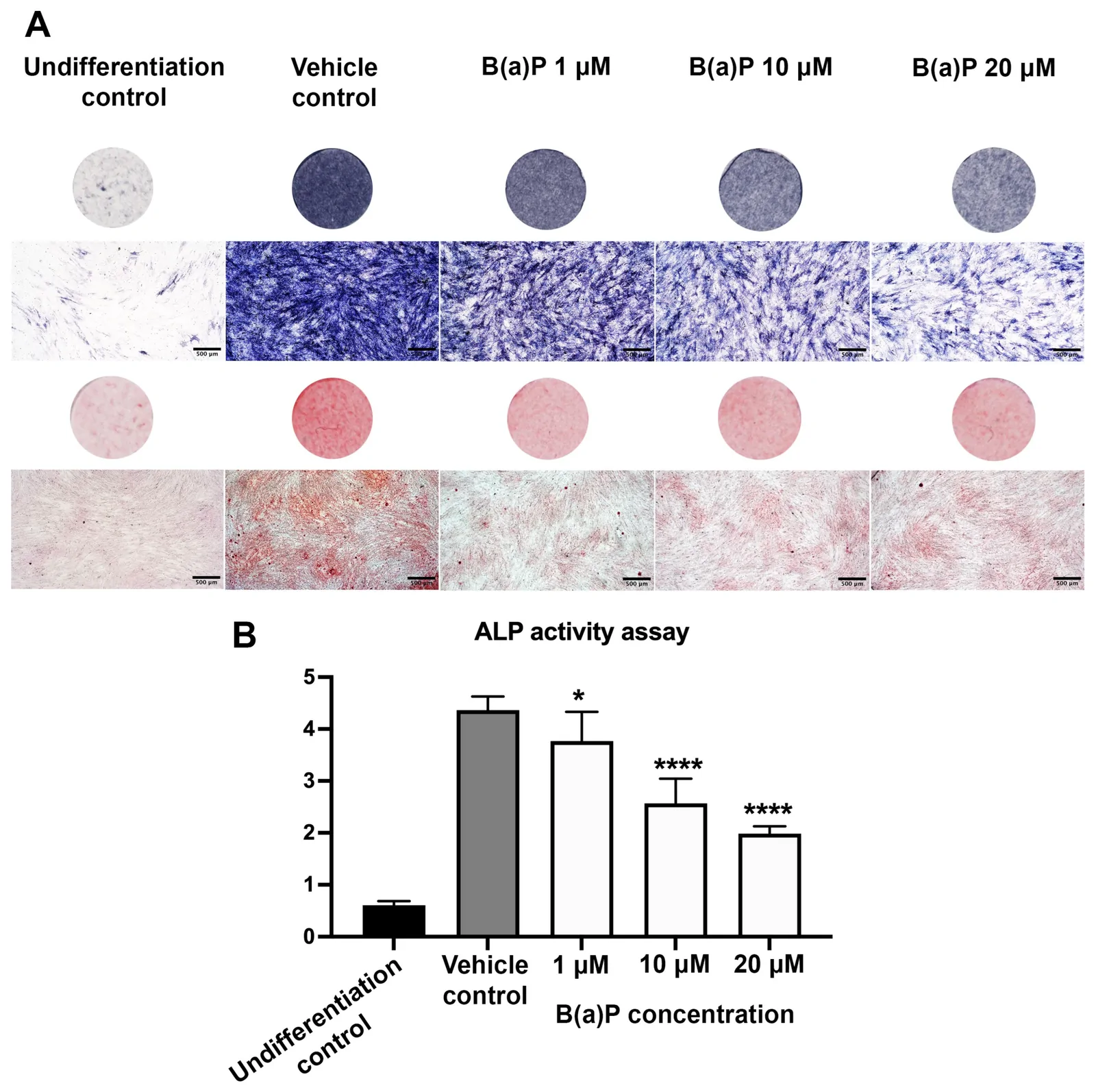
The effect of B(a)P on osteogenic differentiation of BM-hMSCs. (**A**) The effects of B(a)P on osteogenic differentiation were assessed by ALP and Alizarin Red S staining. (**B**) ALP activity after B(a)P treatment under osteogenic differentiation conditions. The data presented as mean  ±  SD (*n* = 3). * *p*  <  0.05, **** *p*  <  0.0001. Scale bar, 500 μm at 4× magnification.

The effect of B(a)P on osteogenic differentiation was examined by ALP assay and Alizarin Red S staining after B(a)P exposures for 14 days under osteogenic induction conditions. ALP staining in differentiated osteoblasts was carried out in order to detect the phenomenon of early osteogenesis. The intensity of ALP staining was reduced in the B(a)P-exposed group compared with the control and differentiation control group (Figure 4A). ALP activity was also measured in B(a)P-exposed cells, revealing a concentration-dependent decrease(Figure 4B). Alizarin Red S staining was performed on differentiated osteoblasts to investigate calcium deposition *in situ*, a markers of terminal osteogenesis. B(a)P at all tested concentrations decreased Alizarin Red S staining intensity compared to the control group (Figure 4A). These results suggest that B(a)P exposure suppressed both early and terminal osteogenic differentiation.

**Figure 5:**
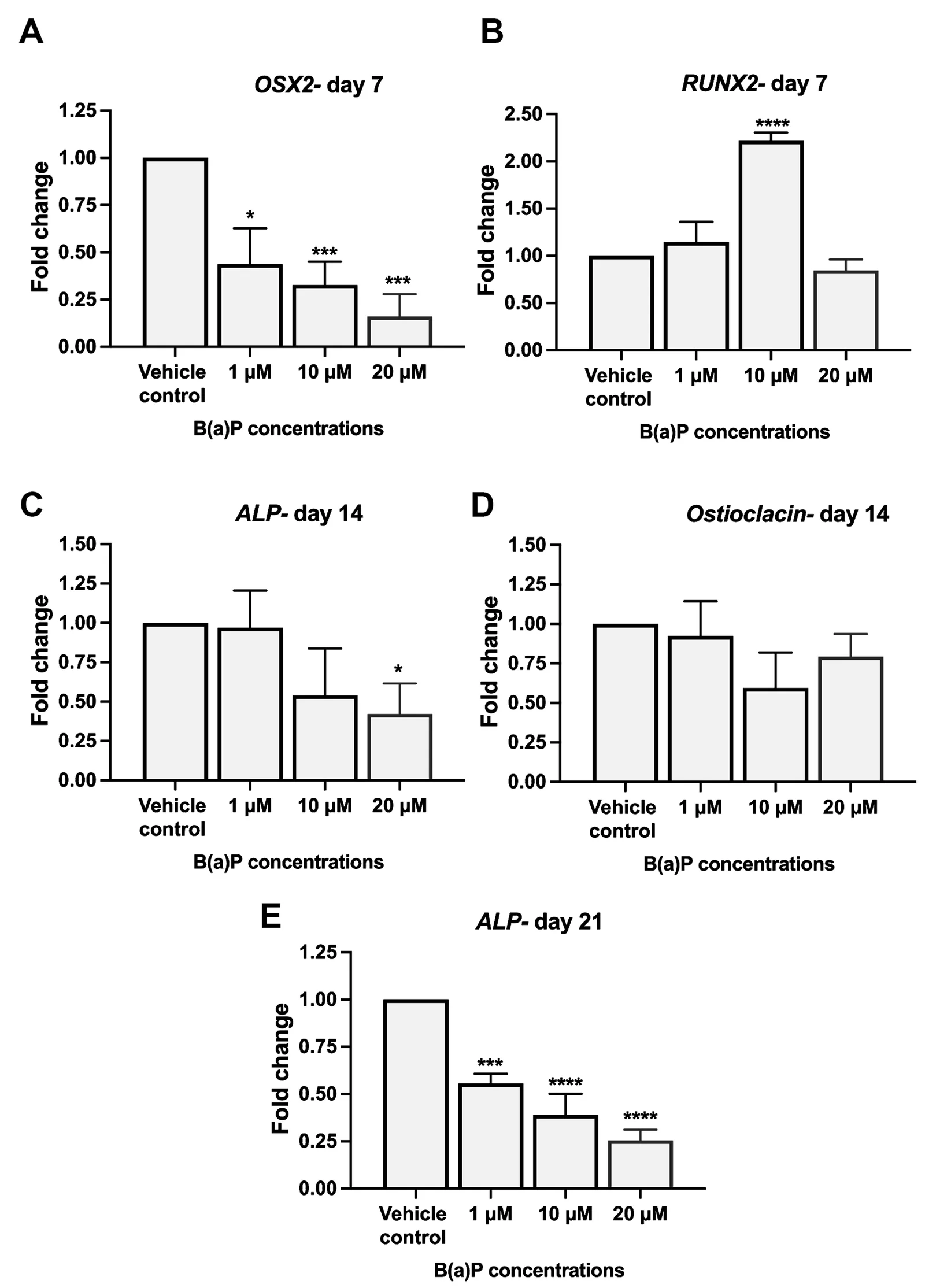
The expression levels of osteogenic differentiation markers in the early phase (7 days: *OSX2* (**A**) and *RUNX2* (**B**)), middle phase (14 days: *ALP* (**C**) and *Osteocalcin* (**D**)), and late phase (21 days: ALP (**E**)) were quantified by qPCR. The data are presented as mean  ±  SD (*n* = 3). * *p*  <  0.05, *** *p*  <  0.001, **** *p*  <  0.0001.

The effect of B(a)P on the expression of osteogenic marker genes *OSX2*, *RUNX2*, *ALP*, and *Osteocalcin* genes were examined using qPCR at 7, 14, and 21 days. After 7 days under osteogenic conditions with B(a)P exposure, the expression levels of transcription factors associated with the early phase of osteogenic differentiation into osteoblast lineage were examined. The mRNA levels of *OSX2* were decreased in all treated concentrations while *RUNX2* was increased at 1 and 10 µM of B(a)P but decreased insignificantly at 20 µM (Figure 5). The middle phase of osteogenic differentiation over 14 days was evaluated for *ALP* and *osteocalcin* mRNA expressions. Exposure to 20 µM of B(a)P significantly decreased *ALP* mRNA expression, while it led to a decreasing trend in *osteocalcin* mRNA expression level. The mRNA expression of *ALP* as the late-phase marker was further significantly suppressed after 21 days of all tested concentrations of B(a)P (Figure 5). These results indicate that B(a)P may impair osteogenic differentiation at all phases in BM-hMSCs.

### 2.5. Expression of Wnt3a and β-Catenin Signaling in B(a)P-Exposed BM-hMSC During Induction of Osteogenic Differentiation

**Figure 6:**
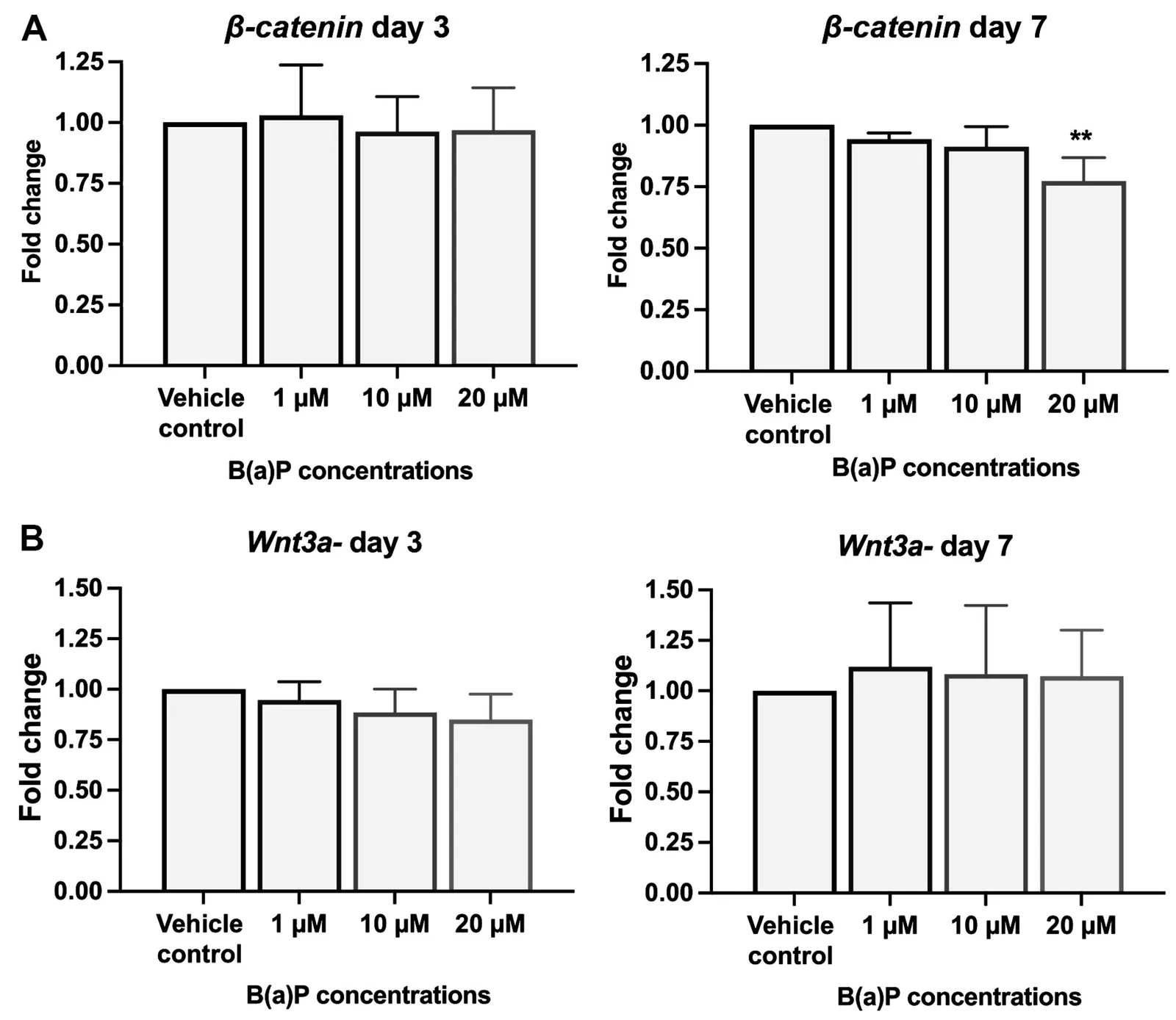
Expression of osteogenic differentiation signaling molecules in B(a)P-exposed BM-hMSCs during osteogenenic differentiation. The expression of β-catenin (**A**) and Wnt3a (**B**) signaling molecules after 3 and 7 days of osteogenic differentiation under B(a)P exposure was analyzed by qPCR. The results were normalized to GAPDH and compared to the untreated vehicle control. ** *p* < 0.01 (*n* = 3).

The mRNA expression of *β-catenin* and *Wnt3a* was analyzed after B(a)P exposure with osteogenic differentiation for 3 and 7 days. *β-catenin* expression was significantly reduced at day 7 following exposure to 20 μM of B(a)P, whereas *Wnt3a* was not significantly reduced at 3 days and remained unchanged after 7 days (Figure 6). These results suggest that B(a)P may alter the expression of *Wnt/β-catenin* signaling molecules during osteogenic differentiation. However, the results may not establish inhibition of Wnt signaling or its effect on proliferation.

### 2.6. Activation of the Aryl Hydrocarbon Receptor (AhR) Following B(a)P Exposure Under Osteogenic Differentiation

**Figure 7:**
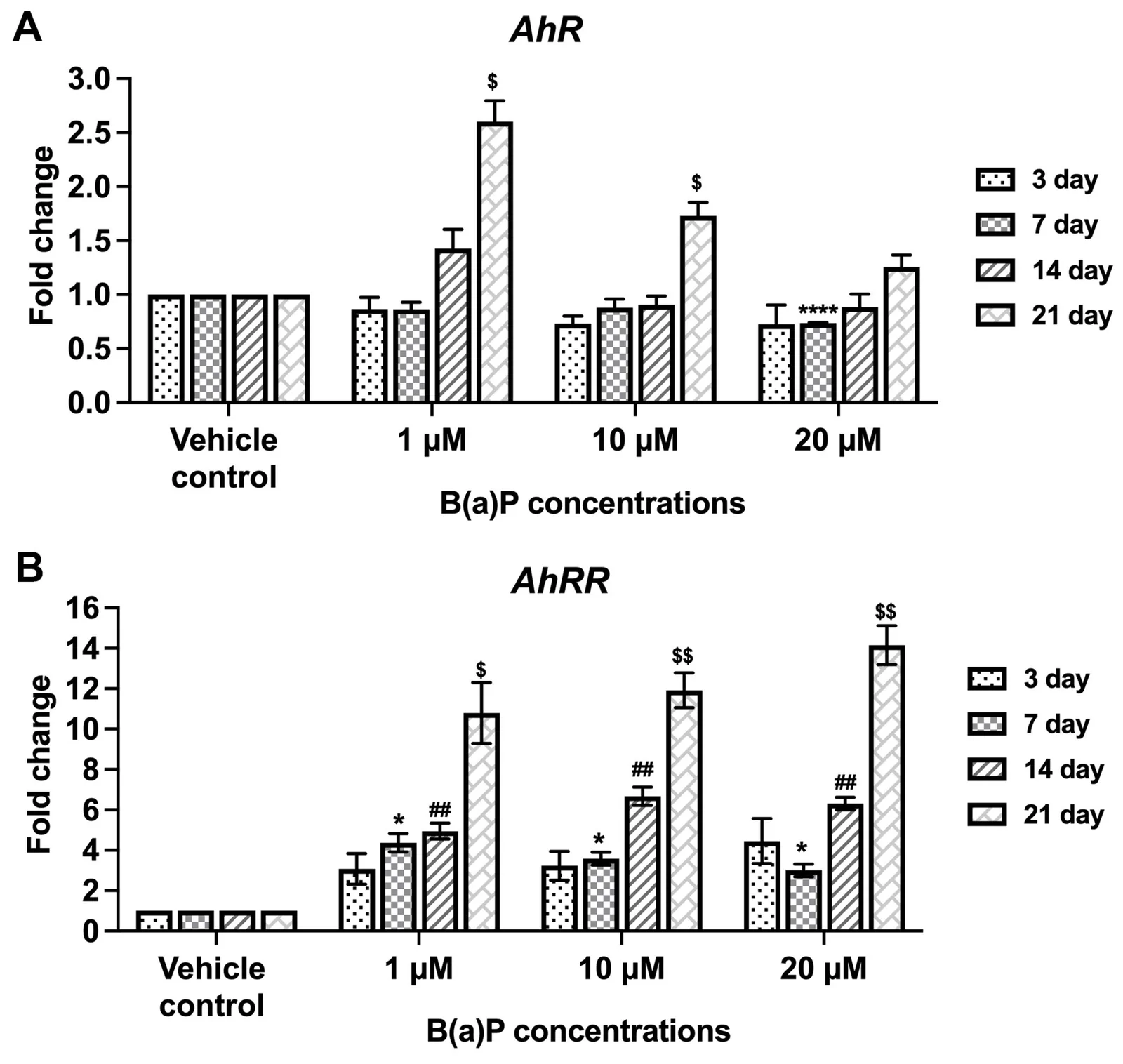
The effect of B(a)P on AhR gene expression. *AhRR* (**A**) and *AhR* (**B**) gene expressions were investigated under osteogenic differentiation conditions using qPCR. Expression was measured at different stages of osteogenic differentiation. Data are expressed as fold changes relative to the control group and presented as mean ± SD (n = 3). Asterisks indicate statistically significant differences between the control and B(a)P-treated groups (1, 10, and 20 µM); 7 (*), 21($) days (ANOVA with Tukey’s multiple comparison test; * *p*  <  0.05, **** *p*  <  0.001, ## *p*  < 0.01, $ *p*  < 0.05, $$ *p*  < 0.01) (*n* = 3).

Following exposure to B(a)P for 3, 7, 14, and 21 days, *AhRR* expression was significantly increased compared with the control group. *AhRR* gene expression was increased in a concentration-dependent manner following exposure to 1–20 µM B(a)P and exhibited a time-course response (Figure 7A), consistent with its role as an AhR-regulated feedback inhibitor.

*AhR* mRNA expression showed the greatest increase at 21 days, with no statistically significant differences between the B(a)P-exposed and vehicle control groups at days 3, 7 and 14 post-exposures compared with a vehicle control (Figure 7B). After 21 days, B(a)P treatment at high concentrations (10–20 µM) promoted AhR expression, but this expression was lower than its expression level at 1 µM of B(a)P exposure, which may reflect ligand-induced receptor downregulation or negative feedback through *AhRR*-mediated pathways that showed concentration-dependent increase. Therefore, BM-hMSCs exposed to B(a)P under osteogenic conditions showed increased *AhR* ligand activity and upregulation of the AhR-responsive transcript AhRR, which may contribute to the inhibition of BM-hMSC differentiation and osteoblast function.

### 2.7. Activation of Senescence-Related Inflammatory Cytokines on B(a)P-Exposed BM-hMSCs During Osteogenesis Differentiation

**Figure 8:**
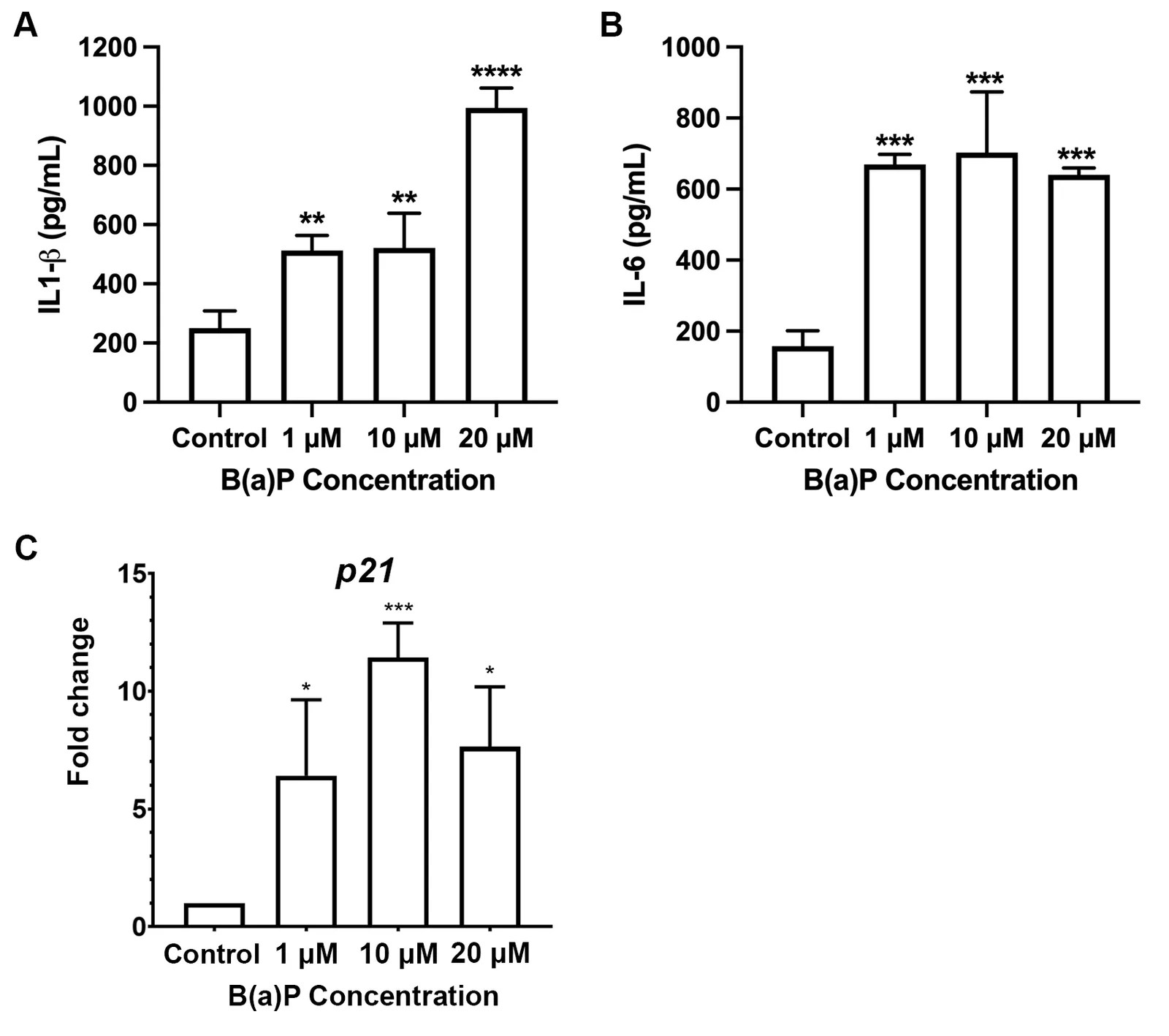
The effect of B(a)P on senescence-related markers. The level of secretory proinflammatory cytokines IL-1β (**A**) and IL-6 (**B**) in the culture media of BM-hMSCs exposed to B(a)P for 14 days under osteogenic differentiation were measured by ELISA. The concentrations of the cytokines were calculated using their respective standard curves. (**C**) Expression of p21 genes during B(a)P exposure together with osteogenic differentiation for 14 days were analyzed by qPCR. Data are presented as mean ± SD (*n* = 3). * *p* < 0.05, ** *p*  <  0.01, *** *p*  <  0.001, **** *p*  <  0.0001 compared to the vehicle control group.

Our results indicated that B(a)P promoted the senescence-associated β-galactosidase phenotypes and increased AhR expression which was associated with inflammation. Thus, we investigated the activation of inflammation-related cytokine following B(a)P exposures under osteogenic differentiation. The levels of proinflammatory cytokines IL-1β and IL-6 in culture supernatants of B(a)P-treated BM-hMSCs cultured in osteogenic induction medium were quantitatively evaluated using ELISA. After 14 days of exposure to 1, 10, and 20 µM of B(a)P, IL-1β and IL-6 levels were higher in all treatment groups than in the vehicle control (Figure 8A,B). The expression of *p16* mRNA level in B(a)P-exposed BM-hMSCs under osteogenic differentiation for 14 days was also analyzed by qPCR. The fold change of p16 mRNA was increased following B(a)P exposure compared with a vehicle control (Figure 8C). These results suggest that B(a)P could induce proinflammatory cytokines and p16 as a senescence marker in BM-hMSCs during osteogenesis differentiation.

### 2.8. Transcriptomic Change After B(a)P Exposure in Osteogenic Differentiation-Induced BM-hMSCs

**Figure 9:**
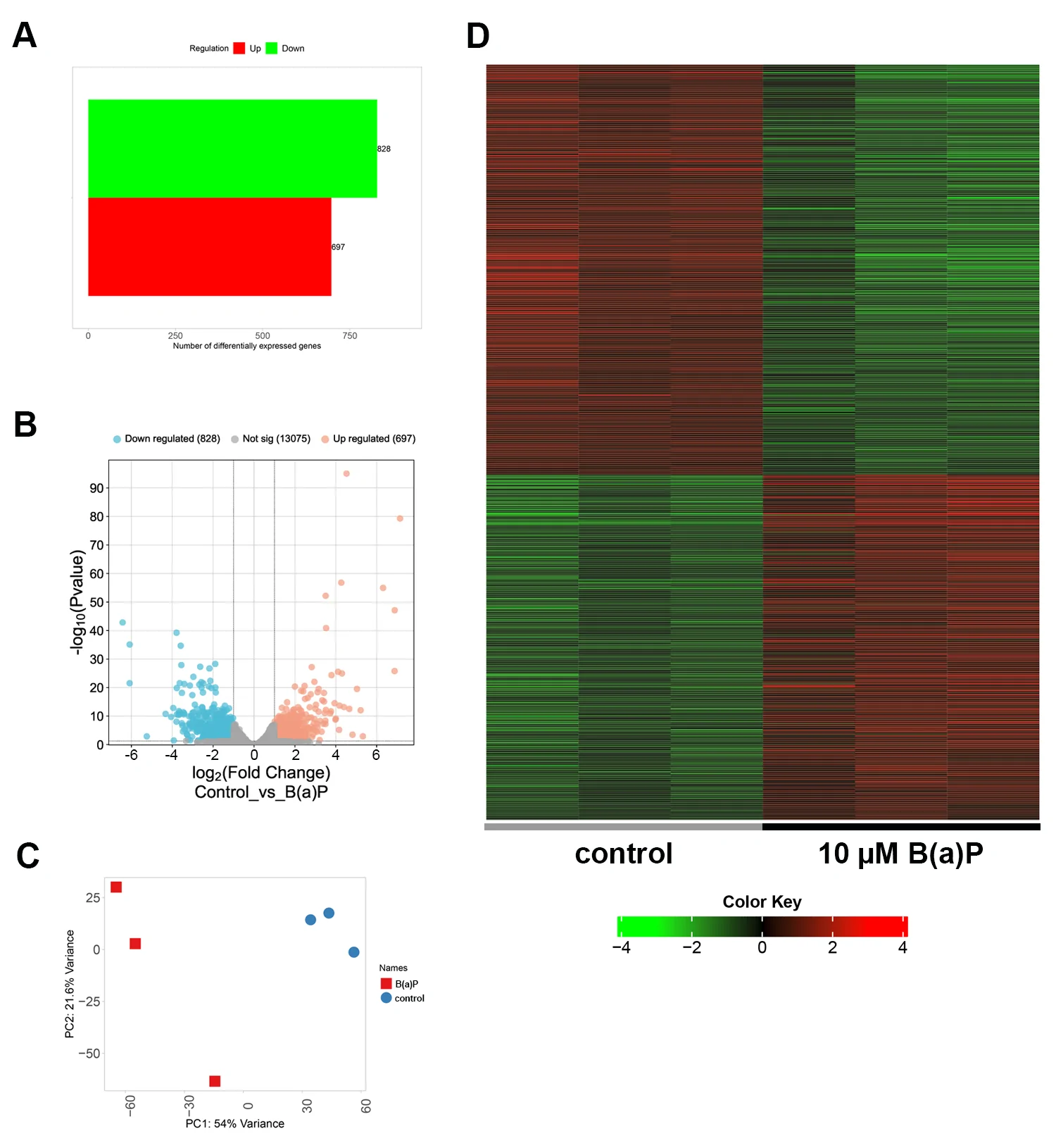
Transcriptomic profiles of BM-hMSCs undergoing osteogenic differentiation following exposure to 10 µM B(a)P for 21 days, as assessed by RNA-seq.. (**A**) Bar graph showing the number of expressed genes in the control and B(a)P-treated groups. (**B**) Volcano plot showing upregulated and downregulated genes following B(a)P exposure. (**C**) PCA plot between control and B(a)P-treated RNA samples. (**D**) Heatmap of normalized read counts revealing differentially expressed genes.

**Figure 10:**
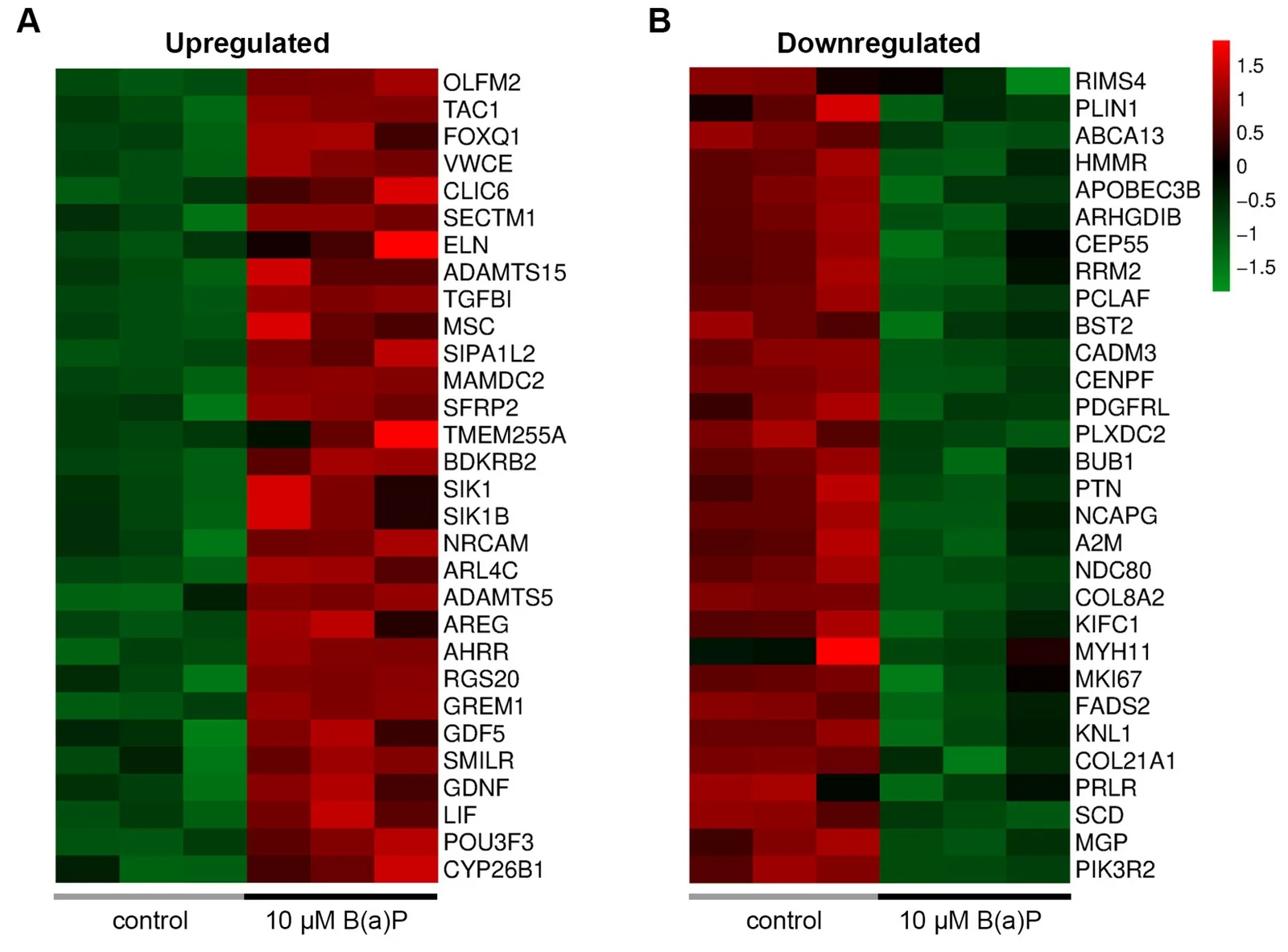
Heatmap of normalized read counts showing the top 30 upregulated (**A**) and downregulated (**B**) genes in BM-hMSCs undergoing osteogenic differentiation following exposure to 10 µM B(a)P for 21 days, compared with vehicle control group.

To identify related gene expression profiles affected by B(a)P exposure in BM-hMSCs, RNA-seq analysis was performed to compare transcriptomic changes between 10 µM B(a)P-exposed and unexposed cells during osteogenic differentiation for 21 days on reference genome *Homo sapiens* GRCh38.81. A total of 697 upregulated and 828 downregulated DEGs were detected between unexposed and B(a)P-exposed groups (Figure 9). The top 30 upregulated and downregulated genes are shown in the heatmap (Figure 10).

Bulk RNA-seq analysis identified molecular pathway interaction associated with B(a)P exposure in BM-hMSCs according to on the Gene Ontology (GO) and Kyoto Encyclopedia of Genes and Genomes (KEGG) databases. Analysis of significant expression of representative genes in the GO pathway of biological processes revealed the downregulation of various biological processes, including the mitotic cell cycle and response to intracellular chemical signals such as those involving DNA primase and ammonium ions. B(a)P exposure was also associated with upregulation of genes involved in stress responses, including apoptosis, trans-synaptic signaling, differentiation of several tissues, dendritic presentation and Wnt signaling (Table 1). B(a)P upregulated representative genes associated with neurotransmitter receptor and glutamate receptors, and downregulated genes encoding structural components, associated with ribosomes, microfibrils, the axon hillock, the presynaptic cytoskeleton, cell adhesion, and lamellar bodies. Genes associated with cellular components involved in cell cycle regulation, including condensin, kinetochore, integrin, DNA replication preinitiation complex and PI3K, were also downregulated in B(a)P-treated cells (Table 2). In the molecular function category of GO, B(a)P-exposed cells revealed upregulation of genes associated with several signaling pathways and enhancers, including vascular endothelial growth factor, protein tyrosine kinase, 14-3-3 protein, MAPK, JNK, CREBP, and transmembrane receptor. It also downregulated representative genes categorized into oxidoreductase, lipoprotein binding, UDP-galactosyltransferase activity, PDGF and peptide hormone binding (Table 3). In the KEGG pathway analysis, B(a)P exposure was associated with upregulation of genes involved in IL-17 signaling, Notch signaling and viral protein interaction with cytokines. Downregulated pathways included cellular metabolism, ribosome, DNA replication and ECM-receptor interaction, and coronavirus disease (Table 4).

**Figure 11:**
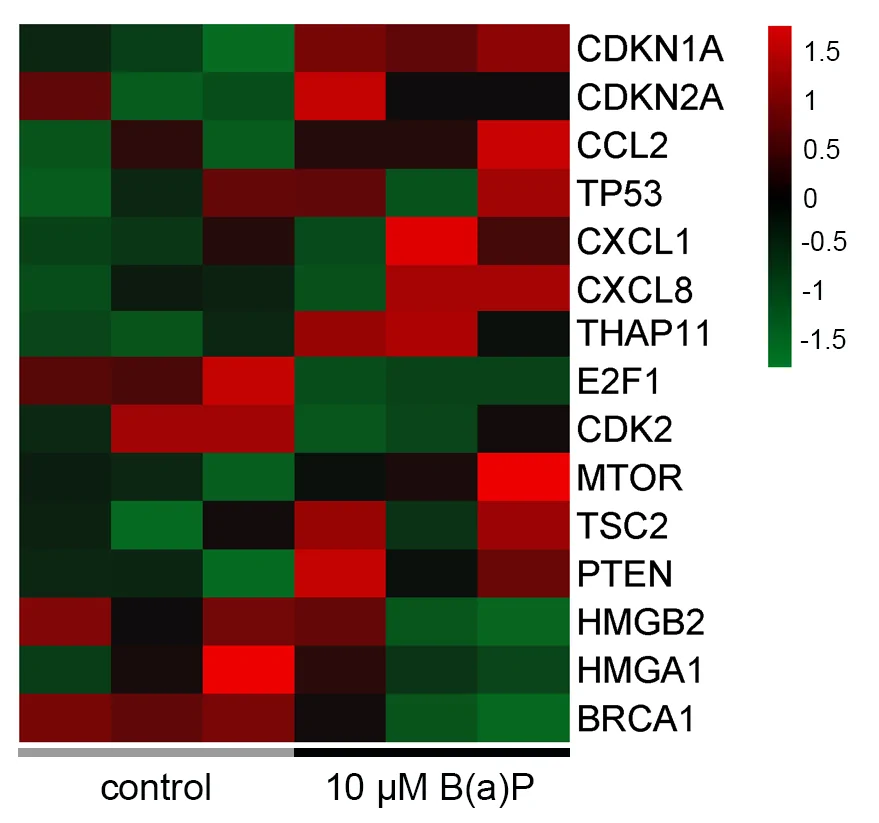
Heatmap of normalized read counts showing differentially expressed senescence and stem cell proliferation-related genes in BM-hMSCs undergoing osteogenic differentiation following exposure at 10 µM B(a)P for 21 days compared with vehicle control group.

Differentially expressed gene profiles of senescence and stem cell proliferation-related genes were also analyzed (Figure 11, Table 5). Classical senescence markers, including *CDKN1A*, *CDKN2A*, *CCL2*, *CXCL1*, and *CXCL8* were upregulated following B(a)P exposure. As referred to in a previous report [18], B(a)P exposure altered expression of representative proliferation and senescence-related genes, including upregulation of *MTOR*, *TSC2*, *PTEN* and *THAP11* and downregulation of CDK2. Transcription factors related to cell proliferation and anti-senescence factors, such as *BRCA1*, *HMGB2* and *E2F1*, were downregulated following B(a)P exposure. Overall, the transcriptomic profile of DEGs implied that B(a)P exposure in BM-hMSCs could downregulate representative genes involved in cell cycle regulation, cellular component integrity and cellular interaction, while upregulating genes associated with stress responses and cytokines.

## 3. Discussion

B(a)P is one of the most extensively studied members of the polycyclic aromatic hydrocarbon (PAH) family and is widely distributed across various environmental pollutants [1,2,3]. Human exposure to PAHs, including B(a)P, poses significant toxicological risks due to their persistence and widespread dispersion in the environment, leading to accumulation in cells and tissues. These compounds are strongly associated with mutagenesis, genotoxicity, immunotoxicity, and developmental toxicity [1,2].

This study demonstrates that B(a)P exposure disrupts the multipotent differentiation capacity of BM-hMSCs, impairs osteogenic and adipogenic differentiation, promotes a senescence-associated phenotype, and activates inflammatory and aryl hydrocarbon receptor (*AhR*)-related signaling pathways. Our findings suggest that B(a)P exposure did not affect toxicity or trigger immediate cell death within 72 h of treatment. However, it induced senescence-associated β-galactosidase (SA-β-Gal) activity in BM-hMSCs under both short-term (48 h) and extended (7-day) exposure conditions, with staining intensity increasing in a time- and concentration-dependent manner. This finding is consistent with previous studies showing that B(a)P inhibits MSC self-renewal and delays fracture healing in vivo [8]. Another study reported that B(a)P suppresses BMP2 signaling via AhR activation [7]. Therefore, our observations provide a more integrated mechanism by linking senescence induction with impaired lineage commitment. The induction of SA-β-Gal activity and senescence markers indicates that MSC aging is a critical determinant of reduced regenerative capacity and highlights that senescent MSCs exhibit reduced osteogenesis and altered secretory profiles [13,19,20]. Mechanistically, senescence-associated impairment of osteogenic differentiation is well documented, with reduced RUNX2 activity and mineralization capacity observed in aged or stress-exposed MSCs [13,21]. B(a)P induces DNA damage and cell cycle arrest through p21 activation and suppression of DNA repair pathways [14,15,22]. Comparable induction of senescence in cancer cells further supports the notion that this response represents a conserved cellular mechanism across different cell types [16]. Our findings extend these observations to BM-hMSCs, suggesting that B(a)P-induced cellular senescence may serve as a key upstream mechanism contributing to impaired MSC differentiation and the disruption of skeletal tissue homeostasis [19]. Importantly, cell viability was assessed only up to 72 h; therefore, we cannot exclude the possibility that prolonged B(a)P exposure affected cell number during the 14- and 21-day differentiation periods. Further studies directly assessing cell viability and cell number throughout the differentiation period are warranted.

Regarding MSC differentiation, B(a)P also exerted an inhibitory effect on adipogenic differentiation, as evidenced by reduced lipid droplet formation, decreased Nile red fluorescence, and downregulation of GLUT4 and adiponectin expression. This anti-adipogenic effect is consistent with previous reports showing that PAHs, via activation of the aryl hydrocarbon receptor (AhR), suppress PPARγ expression and inhibit adipogenic differentiation in human MSCs in an AhR-dependent manner [11,12,23].

Additionally, B(a)P exposure consistently suppressed osteogenic differentiation in BM-hMSCs, as evidenced by reduced ALP activity, diminished matrix mineralization detected by Alizarin Red S staining, and decreased expression of the osteogenic marker genes, including *OSX2*, *ALP*, and OCN at the early, middle, and late phases of differentiation. *OCN* expression showed only a non-significant decreasing trend at day 14 and was not measured at day 21. In contrast, *RUNX2* mRNA expression was increased at 1 and 10 μM at day 7. Because RUNX2 is an early osteogenic transcription factor, this pattern may suggest that early osteogenic commitment was not abolished, whereas progression to osteoblast maturation and mineralization was impaired. The increase in *RUNX2* mRNA does not necessarily reflect increased RUNX2 activity, its protein level and activity would be further examined. These results align with previous reports demonstrating that B(a)P impairs osteoblast differentiation through AhR-mediated suppression of *BMP-2/BMPR-II* signaling [7] and disruption of *TGF-β1/SMAD4* and *ERK/AKT* pathways [8]. Collectively, the consistent inhibitory effects observed in both osteogenic and adipogenic lineages indicate that B(a)P does not simply redirect MSC differentiation toward a particular lineage. Instead, it broadly impairs the differentiation capacity of BM-hMSCs, potentially by driving the cells into a dysfunctional, senescence-associated phenotype. The *Wnt/β-catenin* pathway is focused as the major signaling pathway of osteogenic stem cell proliferation [6]. The reduction in *β-catenin* mRNA at day 7, without corresponding change in *Wnt3a*, suggests that B(a)P may influence Wnt/β-catenin pathway components, but functional evidence of altered pathway activity, such as β-catenin protein or nuclear localization, target gene expression, or reporter activity, was not obtained, and a role of this pathway in the impaired differentiation remains speculative. This possibility is in line with previous studies which reported that AhR-mediated suppression of the BMP and Wnt/β-catenin pathway is a critical regulator of osteoblast differentiation and bone formation, and its suppression has been linked to reduced RUNX2 activation and diminished bone matrix synthesis [6,24,25].

*AhR* was initially characterized as a sensor of environmental toxins, including TCDD and PAH compounds such as B(a)P; however, it is now recognized as a pleiotropic transcription factor that plays critical roles in both developmental processes and age-related pathologies [26,27]. The present findings suggest that B(a)P activates *AhR* in BM-hMSCs, as evidenced by the time- and concentration-dependent upregulation of the *AhR*-responsive target gene *AhRR*. This is consistent with the canonical AhR signaling cascade, wherein ligand-bound AhR translocates to the nucleus, dimerizes with the AhR nuclear translocator (ARNT), and drives transcription of xenobiotic response element (XRE)-containing target genes, including *CYP1A1*, *CYP1B1*, and *AhRR* [9]. Upon induction, *AhRR* acts as a downstream regulator that represses AhR signaling through negative feedback by competing for ARNT binding, thereby reducing AhR transcriptional activity [9]. The progressive increase in *AhRR* expression over 21 days of B(a)P exposure reflects sustained AhR pathway activation. The RNA-seq profile also showed upregulation of *AhRR* expression. Notably, the non-canonical AhR pathway also induces p21CIP1 expression via KLF6 binding to non-consensus XREs [9], providing a direct molecular link between *AhR* activation and the senescence-associated cell cycle arrest observed in this study. In aging biology, AhR signaling displays antagonistic pleiotropy, which is essential for stem cell differentiation and development yet chronic activation later in life may contribute to cellular senescence, osteoporosis, and immune dysregulation upon chronic activation later in life [26,28]. A previous report found that AhR activation promotes oxidative stress, disrupts NAD^+^ metabolism, inhibits autophagy, and accelerates extracellular matrix degradation, which are hallmarks of cellular aging [26]. *AhR* deficiency in aged mice exacerbates cellular senescence, with increased expression of SA-β-gal, p16INK4a, p21CIP1, and SASP factors, including TNF and IL-1 [29]. These findings suggest that the level of AhR pathway activity critically regulates senescence thresholds in both differentiated and progenitor cells. Together, these findings suggest a potential association between AhR pathway engagement, a B(a)P-induced senescence-associated phenotype, and impaired osteogenic differentiation. However, because AhR was not inhibited or knocked down in this study, its direct role in these processes remains to be established and should be tested in future studies.

In the present study, the concurrent induction of SA-β-gal activity and elevation of secretory proinflammatory cytokines IL-1β and IL-6, and *p16* mRNA level under B(a)P-exposed osteogenic conditions, is consistent with this AhR–senescence–SASP axis, in which chronic AhR activation by environmental PAHs may promote a senescence program that is amplifies through autocrine and paracrine inflammatory signaling [30,31]. Together with the upregulation of AhRR, these findings suggest that AhR pathway engagement may contribute to a dysfunctional, senescence-associated phenotype with impaired differentiation capacity in BM-hMSCs under sustained B(a)P exposure. However, the cytokines were measured cumulatively without normalization to viable cell number; a mechanistic role of AhR signaling would be further elucidated. This interpretation is consistent with the literature on the relationship of AhR and bone biology. *AhR* activation reduces osteoblast differentiation and activity, increases bone porosity, and reduces bone matrix mineralization, partly through suppression of *RUNX2* and *ALP* and modulation of Wnt/β-catenin and MAPK signaling cascades. AhR signaling is also more active in cartilage-related diseases such as osteoarthritis, where it further exacerbates endochondral bone formation defects [27,32]. Our finding indicates that B(a)P exposure affects *RUNX2* and *OSX* expression across early stages of differentiation, accompanied by suppression of *ALP* at late stages of differentiation. Together, these results support a model in which sustained *AhR* activation by B(a)P progressively disrupts the transcriptional program required for osteoblast maturation. The increased secretion of IL-1β and IL-6 observed under B(a)P-exposed osteogenic conditions further supports this model, as these SASP-associated cytokines inhibit osteoblastogenesis while promoting osteoclast activation, thereby shifting bone remodeling toward net bone loss [13,33,34].

The transcriptional response to B(a)P exposure in BM-hMSCs undergoing osteogenic differentiation was analyzed by RNA-seq. The mechanism-related transcriptomic profile categorized in both GO and KEGG databases showed that correlation of B(a)P may promote various stress and inflammatory responses with a repressed cell cycle regulation process and perturbed cellular component integrity. Some of the top up- or downregulated DEGs were focused and linked to phenotypic implication of B(a)P-associated responses. *SIK1* (Salt-Inducible Kinase 1) encodes a serine/threonine-protein kinase belonging to the AMPK family. AMPK is recognized as an energy stress sensor in various cell types, including stem cells [35]. A previous study proposed the role of *SIK1* in the promotion of osteoarthritis by binding to 14-3-3, thereby inhibiting of *RUNX2* expression [36]. B(a)P downregulated some marker genes related to cellular signaling and component integrity, such as *PLXDC2* (plexin domain containing 2), which is involved in transmembrane signaling and COL21A1 for collagen formation. In addition, the expressions of senescence-associated genes in MSC following B(a)P exposure was also analyzed by transcriptomic data. The list of senescence-associated genes was referred to a previous report [18]. *HMGB2*, *HMGA1*, and *E2F1*, which are proposed to function as transcription factors associated withcell proliferation and anti-senescence, were downregulated by B(a)P. Together with decreased *CDK2* expression, these findings may indicate cell cycle dysregulation of B(a)P in BM-hMSCs. PI3K-AKT-mTOR signaling and TP53 may act as anti-senescence factors [37]. Downregulation of the PI3K-AKT-mTOR pathway components *MTOR*, together with upregulation of the PI3K-AKT signaling repressors *PTEN* and *TSC2*, in B(a)P-treated cells suggested that senescence induction may occur partly through perturbation of PI3K-AKT-mTOR signaling. Therefore, these results may propose the potential markers for detecting senescence and dysregulated proliferation in MSC by environmental pollutant exposure, and their mechanistic relationship must be explored. In addition, all experiments were performed using the cell line of BM-hMSCs from a single commercial source; therefore, inter-donor variability was not assessed, which may limit the generalizability of the findings.

## 4. Materials and Methods

### 4.1. Cell Culture

BM-hMSCs (PCS-500-012; *Homo sapiens,* human, mesenchymal stem cell from bone) were obtained from the American Type Culture Collection (ATCC, Manassas, VA, USA). Cells were cultured in growth medium consisting of low-glucose Dulbecco’s Modified Eagle Medium (DMEM; Gibco, Thermo Fisher Scientific, Waltham, MA, USA) supplemented with 10% fetal bovine serum (FBS; Gibco, Thermo Fisher Scientific, Waltham, MA, USA), 100 U/mL penicillin, 100 μg/mL streptomycin, and 2 mM of GlutaMAX (Gibco, Thermo Fisher Scientific, Waltham, MA, USA). Cells were maintained at 37 °C in a humidified atmosphere containing 5% CO_2_ until reached confluence for subsequent experiments and used at passages 5–7 for subsequent experiments.

For adipogenic differentiation, the complete high-glucose DMEM medium was supplemented with 0.5 mM of isobutylmethylxanthine (IBMX; Sigma-Aldrich, St. Louis, MO, USA), 0.001 mM of dexamethasone (Sigma-Aldrich, St. Louis, MO, USA), 0.2 mM of indomethacin (Sigma-Aldrich, USA), and 10 μg/mL insulin (Sigma-Aldrich, St. Louis, MO, USA). Osteogenic differentiation was induced using complete low-glucose DMEM supplemented with 100 nM of dexamethasone, 10 mM of β-glycerophosphate, and 50 μg/mL ascorbic acid (Sigma-Aldrich, St. Louis, MO, USA). The culture medium was replaced with a freshly prepared differentiation medium every 3 days.

### 4.2. Cell Viability Assay

BM-hMSCs (1 × 10^4^ cells/well) were plated in 96-well plates overnight. B(a)P (1–50 μM) (Sigma-Aldrich, St. Louis, MO, USA) was treated for 24, 48, and 72 h. The cell viability was determined using the MTT reagent (PanReac AppliChem, Darmstadt, Germany), and the reaction was detected by a microplate reader (Varioskan Flash microplate reader, Thermo Fisher Scientific, Waltham, MA, USA) at the absorbance wavelengths at 570 nm. The percentage of cell viability was calculated by the following equation:

$$\mathrm{Cell viability}\left(\%\right)=\frac{\mathrm{OD}570\;\mathrm{sample}}{\mathrm{OD}570\;\mathrm{Control}}\times 100$$

### 4.3. Senescence-Associated β-Galactosidase (SA-β-Gal) Staining

BM-hMSCs were cultivated overnight at 1.5 × 10^5^ cells/well in 24-well plates. Cells were treated with 20 µM of B(a)P for 48 h and 7 days. Treatment with H_2_O_2_ (Sigma-Aldrich, St. Louis, MO, USA) at 200 µM was used as a positive control at both time points. SA-β-gal activity was detected using SA-β-gal staining Kit (Abbkine, Atlanta, GA, USA). Treated cells were washed with 1×PBS and then fixed with a fixation solution for 15 min at room temperature. After rinsing with 1× PBS at least twice, cells were stained with β-gal staining solution and incubated at 37 °C overnight in an incubator without CO_2_. Under a light microscope (Olympus CKX53, Tokyo, Japan), blue-green granules within the cytoplasm were considered positive for β-gal staining, indicating senescence of the observed cells. Quantitative analysis of SA-β-gal-positive cells was counted, calculated and determined as the fold change relative to the untreated control, which was set to 1.

### 4.4. Adipogenic Differentiation Abilities of BM-hMSCs

BM-hMSCs were used to evaluate their abilities to differentiate into adipocytes by co-treatment with B(a)P. BM-hMSCs (1 × 10^4^ cell/well) were cultured with growth medium until they reached 70−80% confluence. Adipogenic differentiation induction was perfomed using adipogenic induction medium containing DMEM (high-glucose), 10% FBS, 10 μg/mL insulin solution, 0.5 mM of isobutylmethylxanthine, 0.001 mM of dexamethasone, and 0.2 mM of indomethacin (Sigma-Aldrich, St. Louis, MO, USA). B(a)P at final concentrations of 1, 10, and 20 μM was co-treated with an adipogenic induction medium. An undifferentiated control group was maintained in the growth medium. Differentiation-induced cells without B(a)P exposure were defined as the vehicle control. Treated media of all groups were refreshed every 3 days. After culturing for 14 days, adipogenic differentiation was evaluated by Oil Red O staining to detect lipid droplets. Briefly, cells were fixed with 10% formalin vapor for 20 min at room temperature and stained with freshly prepared 0.5% Oil Red O solution (Sigma-Aldrich, St. Louis, MO, USA) for 30 min. Cells containing lipid droplets were captured under an inverted phase contrast microscope (Olympus CKX53, Tokyo, Japan). Additionally, the quantification of adipogenicity differentiation was measured using Nile red and DAPI counterstaining. After being cultured and treated for 14 days, the medium was removed and washed twice with PBS and then fixed with 4% paraformaldehyde for 30 min at room temperature. Subsequently, cells were washed twice with PBS, followed by incubation with DAPI (Sigma; 1:2000 v/v) and Nile red (Sigma; 60 mg/mL) for 20 min. Fluorescent intensity was measured using a microplate reader with DAPI (385/461 nm) and Nile red (553/630 nm) filter sets. Background fluorescence was subtracted with PBS. The Nile red/DAPI ratio was calculated and compared to adipogenic differentiation control group.

### 4.5. Osteogenic Differentiation Abilities of BM-hMSCs

To investigate the effect of B(a)P on osteogenic differentiation in BM-hMSCs, cells were plated in 24-well plates of 4 × 10^4^ cells/well. After the cells reached 80–90% confluence, cells were induced to osteogenic differentiation with osteogenic induction medium consisting of low glucoseDMEM supplemented with 10% FBS, 100 nM of dexamethasone (Sigma-Aldrich, St. Louis, MO, USA), 10 mM of β-glycerophosphate (Sigma-Aldrich, USA), and 50 μg/mL ascorbic acid (Sigma-Aldrich, St. Louis, MO, USA). B(a)P (1, 10, and 20 μM) was co-treated with an osteogenic induction medium, and the cells were cultured for 7, 14, and 21 days. The medium was replaced every 3 days. Differentiated cells without B(a)P treatment were defined as the vehicle control. Phenotypic evaluation of osteogenic differentiation was elucidated by alkaline phosphatase (ALP) staining and Alizarin Red S staining assays.

For the ALP staining assay, after 14 days of incubation, cells were washed with 1×PBS and fixed with 4% paraformaldehyde for 20 min at room temperature. Then, cells were stained with 5-bromo-4-chloro-3-indolylphosphate/nitro blue tetrazolium liquid substrate (BCIP/NBT; Sigma-Aldrich, USA) for 30 min. After washing with deionized water, ALP staining was examined under a light microscope (Olympus CKX53, Tokyo, Japan).

Alizarin Red S staining was performed to detect calcium deposition or mineralized matrix formation during osteogenic differentiation. Briefly, after 14 days of incubation, cells were washed with 1×PBS and fixed with 4% paraformaldehyde for 20 min at room temperature. The cells were then washed with deionized water and stained with 40 mM of Alizarin Red for 30 min at room temperature. Cells were washed with deionized water then observed under a light microscope (Olympus CKX53, Tokyo, Japan).

ALP activity was measured on day 14 after B(a)P treatment using a paranitrophenyl phosphate (pNPP) assay kit (Sigma-Aldrich, St. Louis, MO, USA). Briefly, the cells were incubated with 1% Triton X-100 at room temperature for 20 min. The cell suspension was collected and incubated at 4 °C. The supernatant was mixed with pNPP substrate solution for 15 min, and ALP activity was measured at 405 nm. Total protein concentration was measured using the Bradford assay. ALP activity was calculated with the standard curve generated with the ALP standard solution and normalized with total cellular protein concentration.

### 4.6. qPCR

Gene expression related to adipogenic and osteogenic differentiation was investigated from the effect of B(a)P compounds. Total RNA was extracted and isolated using TRIzol reagent (Invitrogen, Thermo Fisher Scientific, Waltham, MA, USA) and converted to cDNA using iScript select cDNA synthesis kit (Bio-Rad Laboratories, Hercules, CA, USA). Gene expression was then determined by quantitative RT-PCR using the iTaq Universal SYBR Green Supermix Kit (Bio-Rad Laboratories, Hercules, CA, USA), according to the manufacturer’s instructions, with gene-specific primers (Table 6). The results were expressed as fold change in relative mRNA expression, calculated by normalization to GAPDH expression and comparison with the untreated control.

### 4.7. ELISA

Cultured BM-hMSCs were incubated with B(a)P at 1, 10, and 20 μM in osteogenic induction medium for 14 days. The culture supernatant was collected at each medium change and pooled to represent cumulative secretion over 14 days and then concentrated by Amicon Ultra-4 Centrifugal Filter 3 kDa (Merck, Rahway, NJ, USA). The secretion of proinflammatory cytokines, including IL1-β and IL-6, was detected using an homogenous time-resolved fluorescence (HTRF)-ELISA kit (Revvity, Waltham, MA, USA) according to the manufacturer’s instructions. The reactions were observed by a multimode plate reader at 665 and 620 nm (Revvity, Waltham, MA, USA). Cytokine concentrations were calculated using the standard curve for each cytokine and expressed as pg/mL equivalents.

### 4.8. Transcriptomic Analysis

Total RNA was prepared for RNA-seq analysis from BM-MSCs cultured under two experimental conditions:an unexposed control group (n = 3) and a group exposed to 10 µM of B(a)P (n = 3), both undergoing osteogenic differentiation for 21 days. Transcriptomic analysis was performed by National Omics Center, the National Science and Technology Development Agency (NSTDA), Thailand. RNA libraries were prepared with the MGIEasy RNA Library Prep Set according to the manufacturer’s instructions and sequenced on the MGISEQ-2000RS platform (MGI Tech, Shenzhen,China). Sequencing reads were aligned to the human reference genome (Homo sapiens GRCh38.81) using HISAT2 (version 2.2.1) [38], followed by transcript assembly and expression quantification with StringTie (version 1.3.6). Initial differential expression analysis was performed using the Ballgown R package [39], and the gene-level read count matrix was subsequently analyzed using the Integrated Differential Expression and Pathway Analysis (iDEP) pipeline (version 2.4.3) [40]. Genes were filtered using a minimum threshold of 0.5 counts per million (CPM) in at least five libraries, and principal component analysis (PCA) was conducted to assess global expression patterns and the clustering of biological replicates. Differentially expressed genes (DEGs) were identified using the DESeq2 algorithm with a false discovery rate (FDR) < 0.05 and an absolute fold change ≥ 2. Functional enrichment analysis of DEGs was performed using the Kyoto Encyclopedia of Genes and Genomes (KEGG) and Gene Ontology (GO) databases, including Biological Process, Cellular Component, and Molecular Function categories, with statistical significance defined as an FDR-adjusted *p*-value < 0.05.

### 4.9. Statistical Analysis

Data are presented as mean ± SD from at least three independent experiments. Statistical analysis was performed using GraphPad Prism software version 10 (GraphPad Software, CA, USA) with one-way ANOVA followed by Dunnett’s post hoc test or two-way ANOVA Tukey’s multiple comparison test. Statistical significance was indicated as follows: * indicates *p* < 0.05; ** indicates *p* < 0.01; *** indicates *p* < 0.001, and **** indicates *p* < 0.0001.

## 5. Conclusions

This study proves that molecular correlation of B(a)P exposure orchestrates an AhR–senescence–inflammation axis in differentiated BM-hMSCs. Collectively, B(a)P may impair their multipotent differentiation capacity, and the study highlights relevant implications for skeletal health risk associated with exposure to environmental pollutants. This study has certain limitations that should be acknowledged. Senescence-related effects of B(a)P in this study were described only by ELISA detection of secretory senescence-associated markers and transcriptomic profiles, which may not be sufficient to reach a general conclusion. Molecular screening and the mechanistic perspective of the senescence effect should be further investigated. Mechanistic investigation of signaling pathways mediating MSC senescence induced by B(a)P and other related combined substances should be carried out.

## Figures and Tables

**Table 1:** GO pathway of biological process.

Direction	Pathways	Genes	*p* Value
Down	Meiotic cell cycle phase transition	5	1.40 × 10^−2^
	Response to bacterial lipoprotein	5	1.60 × 10^−2^
	Regulation of DNA primase activity	5	2.70 × 10^−2^
	Positive regulation of DNA primase activity	5	2.70 × 10^−2^
	Response to ammonium ion	5	4.80 × 10^−2^
Up	Regulation of Wnt signaling pathway involved in heart development	5	2.10 × 10^−4^
	Neuroendocrine cell differentiation	6	6.60 × 10^−4^
	Regulation of intrinsic apoptotic signaling pathway in response to osmotic stress	5	2.90 × 10^−3^
	Wnt signaling pathway involved in heart development	6	4.50 × 10^−3^
	Cardiac vascular smooth muscle cell differentiation	6	5.00 × 10^−3^
	Retrograde trans-synaptic signaling by lipid	5	6.80 × 10^−3^
	Retrograde trans-synaptic signaling by endocannabinoid	5	6.80 × 10^−3^
	Regulation of epithelial tube formation	6	9.10 × 10^−3^
	Positive regulation of epithelial tube formation	6	9.10 × 10^−3^
	Dendritic cell antigen processing and presentation	6	1.00 × 10^−2^
	Regulation of inner ear auditory receptor cell differentiation	5	1.80 × 10^−2^
	Regulation of mechanoreceptor differentiation	5	1.80 × 10^−2^
	Regulation of inner ear receptor cell differentiation	5	1.80 × 10^−2^
	Regulation of cerebellar granule cell precursor proliferation	5	1.90 × 10^−2^
	Regulation of dendritic cell antigen processing and presentation	5	4.00 × 10^−2^

**Table 2:** GO pathway of cellular component.

Direction	Pathways	Genes	*p* Value
Down	Cytosolic large ribosomal subunit	53	1.10 × 10^−7^
	Outer kinetochore	16	1.20 × 10^−4^
	Presynaptic cytoskeleton	6	1.80 × 10^−3^
	Protein complex involved in cell adhesion	37	2.50 × 10^−3^
	Integrin complex	22	4.30 × 10^−3^
	Inner kinetochore	18	9.50 × 10^−3^
	Axon hillock	6	1.20 × 10^−2^
	Phosphatidylinositol 3-kinase complex class ia	6	1.70 × 10^−2^
	Phosphatidylinositol 3-kinase complex class i	6	1.70 × 10^−2^
	Cmg complex	10	2.40 × 10^−2^
	Microfibril	10	2.50 × 10^−2^
	Dna replication preinitiation complex	11	2.50 × 10^−2^
	Cytoskeleton of presynaptic active zone	5	2.70 × 10^−2^
	Juxtaparanode region of axon	7	3.60 × 10^−2^
	Condensin complex	8	3.60 × 10^−2^
	Lateral element	11	3.60 × 10^−2^
	Lamellar body	6	5.00 × 10^−2^
Up	Ionotropic glutamate receptor complex	15	3.80 × 10^−2^
	Neurotransmitter receptor complex	15	3.80 × 10^−2^
	Ampa glutamate receptor complex	11	4.60 × 10^−2^

**Table 3:** GO pathway of molecular function.

Direction	Pathways	Genes	*p* Value
Down	Low-density lipoprotein particle receptor activity	10	1.00 × 10^−2^
	Galactosyltransferase activity	22	1.00 × 10^−2^
	Oxidoreductase activity acting on paired donors with oxidation of a pair of donors resulting in the reduction of molecular oxygen to two molecules of water	8	1.90 × 10^−2^
	Lipoprotein particle binding	17	2.10 × 10^−2^
	Protein–lipid complex binding	17	2.10 × 10^−2^
	Udp-galactosyltransferase activity	19	2.20 × 10^−2^
	Lipoprotein particle receptor activity	11	3.60 × 10^−2^
	Peptide hormone binding	19	3.80 × 10^−2^
	Platelet-derived growth factor binding	9	4.70 × 10^−2^
	Beta-1,3-galactosyltransferase activity	9	4.70 × 10^−2^
Up	Vascular endothelial growth factor receptor binding	10	3.90 × 10^−2^
	Heparan sulfate sulfotransferase activity	8	2.70 × 10^−3^
	Protein tyrosine kinase activator activity	15	3.90 × 10^−3^
	14-3-3 protein binding	29	3.90 × 10^−3^
	Transmembrane receptor protein tyrosine kinase activator activity	9	4.00 × 10^−3^
	Vascular endothelial growth factor receptor 2 binding	8	4.40 × 10^−3^
	Mitogen-activated protein kinase kinase binding	11	7.80 × 10^−3^
	Camp response element binding protein binding	10	1.10 × 10^−2^
	Jun kinase binding	10	2.20 × 10^−2^
	Camp response element binding	12	4.70 × 10^−2^

**Table 4:** KEGG pathway.

Direction	Pathways	Genes	*p* Value
Down	Coronavirus disease, COVID-19	153	5.70 × 10^−7^
	Ribosome	121	1.90 × 10^−6^
	Steroid biosynthesis	14	6.60 × 10^−4^
	Progesterone-mediated oocyte maturation	76	7.70 × 10^−4^
	Dilated cardiomyopathy	55	1.10 × 10^−3^
	Terpenoid backbone biosynthesis	19	3.40 × 10^−3^
	Arrhythmogenic right ventricular cardiomyopathy	45	3.40 × 10^−3^
	Hypertrophic cardiomyopathy	54	4.90 × 10^−3^
	Biosynthesis of unsaturated fatty acids	21	5.30 × 10^−3^
	ABC transporters	26	5.70 × 10^−3^
	PPAR signaling pathway	45	7.00 × 10^−3^
	Fatty acid metabolism	45	7.90 × 10^−3^
	ECM–receptor interaction	52	1.80 × 10^−2^
	Butanoate metabolism	15	1.90 × 10^−2^
	Valine leucine and isoleucine degradation	41	2.50 × 10^−2^
	N-Glycan biosynthesis	41	4.00 × 10^−2^
	DNA replication	32	4.10 × 10^−2^
Up	IL-17 signaling pathway	55	1.10 × 10^−3^
	Notch signaling pathway	42	1.40 × 10^−2^
	Viral protein interaction with cytokine and cytokine receptor	25	5.00 × 10^−2^

**Table 5:** Differentially expressed level of selected senescence and stem cell proliferation-related genes.

Genes	Ensembl ID	Log2 Fold Change
CDKN1A	ENSG00000124762	2.311096969
CDKN2A	ENSG00000147889	0.232184542
CCL2	ENSG00000108691	0.650092199
TP53	ENSG00000141510	0.149611308
CXCL1	ENSG00000163739	0.882841862
CXCL8	ENSG00000169429	0.5808492
THAP11	ENSG00000168286	0.583023551
E2F1	ENSG00000101412	−2.087759724
CDK2	ENSG00000123374	−0.584441897
MTOR	ENSG00000198793	0.278903258
TSC2	ENSG00000103197	0.161890459
PTEN	ENSG00000171862	0.387985631
HMGB2	ENSG00000164104	−0.618176732
E2F1	ENSG00000101412	−2.087759724
BRCA1	ENSG00000012048	−1.291656635
HMGA1	ENSG00000137309	−0.781042319
CDKN1A	ENSG00000124762	2.311096969
CDKN2A	ENSG00000147889	0.232184542
CCL2	ENSG00000108691	0.650092199

**Table 6:** List of primers.

Primer	Forward Primer 5′-3′	Reverse Primer 5′-3′
Adipocyte Marker		
Adiponectin	CCTGGTGAGAAGGGTGAGAA	CAATCCCACACTGAATGCTG
GLUT4	CTTCGAGACAGCAGGGGTAG	ACAGTCATCAGGATGGCACA
Osteogenic differentiation		
OSX2	GCCAGAAGCTGTGAAACCTC	GCTGCAAGCTCTCCATAACC
RUNX2	CCTCGGAGAGGTACCAGATG	TTCCCGAGGTCCATCTACTG
ALP	CCTTGCTCACTCACTCACTCC	TTTTTTTTGCCGTTCCAAAC
Osteocalcin	GTGCAGAGTCCAGCAAAGGT	TCAGCCAACTCGTCACAGTC
Proliferation markers		
β-catenin	GCTTGTTCGTGCACATCAGGA	TGTGAACATCCCGAGCTAGGA
WNT3a	ATGAACCGCCACAACAACGAGG	GTCCTTGAGGAAGTCACCGATG
AhR receptor		
Ahr	ACATCACCTACGCCAGTCG	CGCTTGGAAGGATTTGACTTG
Ahrr	CTTAATGGCTTTGCTCTGGTCG	TGCATTACATCCGTCTGATGGA
Senescence marker		
P21	KiCqStart™ SYBR Green Primers for human CDKN1A (Merck, Rahway, NJ, USA), Cat#H_CDKN1A_2	

## Data Availability

The original contributions presented in this study are included in the article. Further inquiries can be directed to the corresponding authors.
